# Contribution of the EuroRotaNet surveillance network to rotavirus strain surveillance in Europe

**DOI:** 10.2807/1560-7917.ES.2025.30.38.2400798

**Published:** 2025-09-25

**Authors:** Natasha Marcella Vaselli, Miren Iturriza-Gómara, Daniel Hungerford, EuroRotaNet network members, EuroRotaNet network members, Stephan Aberle, Jelle Matthijnssens, Marc Van Ranst, Sofie Midgley, Kristina T Franck, Elizabeth Tatsi, Vassiliki P Syriopoulou, Dimitra-Maria Koukou, Krisztián Bányai, Renáta Dóró, Jérôme Kaplon, Alexis de Rougemont, Carita Savolainen-Kopra, Haider Al-Hello, Franco Maria Ruggeri, Giovanni Ianiro, Marina Monini, Tina Triglav, Tina Mikuletic, Javier Buesa, Gustavo Cilla, Milagrosa Montes, Lena Dillner, Lottie Schloss, Klas Strååt, Andreas Mas Marques, Cristina Celma, Stuart Beard

**Affiliations:** 1 The Centre for Global Vaccine Research, Institute of Infection, Veterinary and Ecological Sciences, University of Liverpool, Liverpool, United Kingdom; 2 National Institute for Health and Care Research (NIHR) Health Protection Research Unit in Gastrointestinal Infections, Liverpool, United Kingdom; 3 The members of the networks are listed under Collaborators

**Keywords:** Rotavirus, diarrhoea, surveillance, genotypes, strains, vaccine

## Abstract

Two rotavirus strain surveillance networks operate in Europe: the World Health Organization Global Rotavirus Surveillance Network and the European Rotavirus Network (EuroRotaNet). We describe and appraise rotavirus strain surveillance, with a focus on EuroRotaNet, which has conducted rotavirus strain surveillance since 2007 across 21 European countries. Since EuroRotaNet began, epidemiological and genotype data has been collected on over 90,000 rotavirus-positive specimens. We identified distinct differences in strain distribution across seasons, countries, regions and age cohorts. As infant rotavirus vaccination has been rolled out in some European countries, the EuroRotaNet surveillance network is able to monitor changes in strain type circulation and signs of potential emergence of vaccine escape strains, comparing countries with and without vaccination programs. Despite natural fluctuations in strain distribution, the data show an increase in strain diversity after vaccine introduction, although no strain displacement due to vaccination nor emergence of unusual strains of epidemiologically significance were noted. The EuroRotaNet surveillance network takes a pragmatic approach to surveillance and is not overly prescriptive, creating a wide, engaged and sustainable network.

## Background

Rotavirus infection is the leading cause of severe diarrhoea in children aged younger than 5 years, globally [1]. It is primarily transmitted by the faecal-oral route, with symptoms typically developing 1–2 days following infection [2]. Symptoms include nausea, vomiting, diarrhoea, abdominal pain, fever and severe dehydration which can lead to death. By the age of 3–5 years, almost every child has been infected with rotavirus. Most deaths occur in low-middle income countries [1,2].

There are nine rotavirus species, A–J, defined by the middle VP6 capsid antigen. In humans, the majority of infections are caused by rotavirus species alphagastroenteritidis (formerly known as rotavirus A), as defined by the middle capsid viral protein VP6 [3]. Nucleotide sequences of the two outer capsid proteins, VP7 (a glycoprotein) and VP4 (a protease sensitive protein), are the basis for the dual classification system of rotavirus, defining G and P types, respectively [4]. Whole genomic sequencing has allowed specific genotypes to be assigned to each of the 11 genome segments which make up the whole genome of a particular rotavirus strain. Rotavirus A may also be characterised to different genomic constellations, and the majority of viruses causing human disease belong the Wa-like (G1/G3/G4/G9-P[8]-I1-R1-C1-M1-A1-N1-T1-E1-H1) or the DS-1-like (G2P[4]-I2-R2-C2-M2-A2-N2-T2-E2-H2) genotype-constellation [4].

In 2006, the rotavirus vaccines Rotarix (GlaxoSmithKline Biologicals, Wavre, Belgium) and RotaTeq (Merck and Co., Inc, Rahway, United States) were recommended for use in Europe and the Americas (Table 1) [5]. In 2009, the World Health Organization (WHO) Strategic Advisory Group of Experts on Immunization (SAGE) recommended the inclusion of rotavirus vaccination of infants into all national immunisation programmes after clinical trials in low- and middle-income countries [5]. In 2020, SAGE recommended the worldwide use of the Rotavac (Bharat Biotech Ltd., Hyderabad, India) and Rotasiil (Serum Institute of India Ltd., Pune, India) rotavirus vaccines, which were internationally prequalified for use by the WHO and were initially licensed in India [6].

**Table 1:** Rotavirus vaccines pre-qualified by the World Health Organization

Vaccine	Vaccine type	Composition	Recommended schedule
Rotarix (GlaxoSmithKline Biologicals)	Oral live attenuated	Human rotavirus vaccine derived from the circulating human wild‐type rotavirus strain G1P[8].	Two oral doses started before 16 weeks of age and completed before 24 weeks of age.
Rotavac (Bharat Biotech Ltd.)	Oral live attenuated	A vaccine derived from naturally occurring human reassortant G9P[11] strain: 116E.	Three doses are recommended, administered at 6, 10 and 14 weeks of age.
RotaTeq (Merck and Co., Inc.)	Oral, live, human- bovine reassortant vaccine	Multivalent vaccine containing five live, human-bovine reassortant rotavirus strains. Four reassortant rotavirus strains express one of the common human VP7 (G) genotypes; G1, G2, G3, and G4, and the fifth reassortant expresses human VP4 (P) genotype P[8].	Three doses are recommended; administered between 6 and 32 weeks of age, with the first dose given at 6 to 12 weeks of age and subsequent doses administered at 4 to 10-week intervals.
Rotasiil (Serum Institute of India Ltd.)	Oral, live, human- bovine reassortant vaccine	Multivalent vaccine containing five live, human-bovine reassortant rotavirus strains possessing the following human rotavirus genotypes; G1, G2, G3, G4, and G9.	Three doses are recommended, to be administered at 6, 10 and 14 weeks of age.

Rotavirus vaccines have shown to be highly efficacious and effective against severe rotavirus disease in children under 5 years of age [2]. However, their effectiveness varies by setting, being highest in high-income settings and lowest in low-middle income countries [2]. There has also been substantial evidence of indirect protective effects of rotavirus vaccination, including in older children [7]. A recent systematic review estimated that global mortality from rotavirus gastroenteritis in children younger than 5 years has fallen from estimates of 316,634 per year in 2000 to 108,322 per year in 2021 [8].

**Figure 1:**
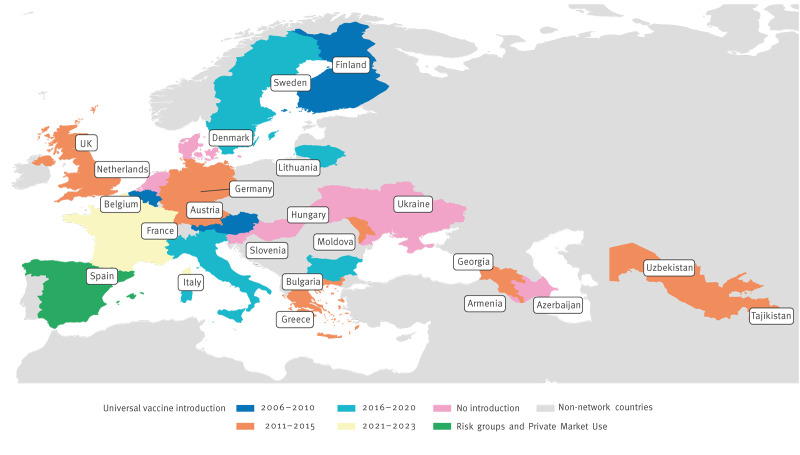
Vaccine introduction for rotavirus strain surveillance network countries, Europe, 2023

In 2023, 30 of 53 countries in the WHO European Region had introduced routine rotavirus vaccination (either Rotarix and/or RotaTeq) into their infant immunisation schedules either regionally (n = 2), in risk groups (n = 3) or universally (n = 25) [9,10]. Several countries, such as Austria, Belgium, Finland, Germany, Sweden and the United Kingdom (UK), achieved high rotavirus vaccination coverage (> 80%) [9] (Figure 1). In other European countries, rotavirus vaccine access is more variable with different national authority recommendations and financial support/reimbursement schemes. In 2022, the French National Authority for Health recommended rotavirus vaccination as part of the national immunisation programme with full reimbursement [11]. However, many European countries have no national authority recommendation for rotavirus vaccination and have an estimated coverage of < 10% in the eligible population [9].

Data adapted from [35,36].

With the roll out of rotavirus vaccination programmes, it is conceivable that immune pressure could drive the emergence of vaccine escape mutants. However, even in countries without vaccination, changes in genomic regions of interest for neutralising rotaviruses have been observed [12]. Several investigations have highlighted that the evolution of rotavirus strains by intra- or inter-genotype reassortment or mutation is a relatively common phenomenon that may also involve gene donation by vaccine-derived strains [13]. Natural infection and vaccination elicit both homotypic and heterotypic antibody responses [14]. Studies using mathematical models have predicted that small differences in strain-specific vaccine effectiveness may lead to strain selection, but this could take years to become apparent [15].

Rotavirus surveillance systems were introduced to monitor the trends of rotavirus genotypes, the emergence of new rotavirus strains and the impact of rotavirus vaccines. To date, there are two main rotavirus surveillance systems in Europe: the European Rotavirus Network (EuroRotaNet, https://www.eurorotanet.com/) and the WHO Global Rotavirus Surveillance Network (GRSN). In this perspective, we focus on describing and appraising EuroRotaNet in order to evidence the importance and challenges associated with collaborative, pragmatic, low-cost cross border-surveillance networks for vaccine preventable diseases.

## Description of the European Rotavirus Network

The EuroRotaNet surveillance network was established in January 2007 to gather information on the rotavirus genotypes co-circulating throughout Europe pre- and post- vaccine introduction [16]. It consists of a voluntary network of European laboratories, and network activities are funded between the collaborating institutes and industry. For the past 18 years, EuroRotaNet has conducted rotavirus strain surveillance in Europe and includes data dating back to September 2005. The aims of EuroRotaNet are detailed in the Box.

Box **Aims of the EuroRotaNet**

- Develop and maintain methods for effective rotavirus typing and characterisation;
- Monitor the effectiveness of current genotyping methods and respond to changes associated with genetic drift and shift;
- Describe in detail the molecular epidemiology of rotavirus infections in Europe, during consecutive rotavirus seasons, through genotyping of rotavirus-positive samples;
- Monitor the emergence and spread of common and novel rotavirus strains within Europe;
- Develop the infrastructure that may serve as a platform for additional surveillance activities and nested studies for evaluating the impact of a rotavirus vaccine in the general population; the possible emergence in the general population of genotypes other than those included in the vaccine; and the possible emergence in the general population of reassortants between vaccine and naturally circulating wild-type strains [17,19].

Only samples identified by routine diagnostic testing as rotavirus-positive are included in EuroRotaNet. The inclusion criteria for specimens are faecal samples collected during an episode of acute gastroenteritis and a maximum of two samples from an outbreak. While there are no age cut-off criteria, this is dependent on country-specific clinical diagnostic pathways. The faecal samples are subsequently typed within each country by EuroRotaNet laboratories using standardised PCR-based G and P typing methods, previously described and available at eurorotanet.com. A country-level minimum target number of specimens included for genotyping has been set at 50–60. These targets enable detection of rotavirus genotypes with a prevalence of ≥ 1% in countries without universal rotavirus vaccination. Partner laboratories in member countries then submit typing results linked to epidemiological case data to the EuroRotaNet database. These data contain details on hospitalised and community cases of any age, collected from rural and urban populations [16,17].

The EuroRotaNet surveillance network has been able to collect data from a total of 21 European countries, 16 of which are or were member countries [16]. Denmark, Finland, France, Germany, Hungary, Italy, Slovenia, Spain, Sweden and the UK joined in 2007. Belgium joined in January 2008, Greece in January 2009 and Austria in December 2010. Bulgaria and Lithuania were members of EuroRotaNet from January 2008 until August 2013 and the Netherlands from 2007 until August 2017 [18]. The EuroRotaNet surveillance network currently includes 13 member countries (Table 2).

**Table 2:** Member countries included in the EuroRotaNet European Rotavirus Network, 2007–2023^a^ [17]

Country	Vaccine introduction into paediatric immunisation programme	Vaccine used	Most recent coverage data^b^	Reimbursement (%)	Surveillance years
Austria	2006	Rotarix/RotaTeq	High	100	From 2011
Belgium	2007	Rotarix/RotaTeq	High	Partial	From 2008
Bulgaria	2017	Rotarix/RotaTeq	Unknown	100	2008–2013
Denmark	None	NA	NA	NA	From 2007
Finland	2009	RotaTeq	High	100	From 2007
France	2022	Rotarix/RotaTeq	Low	100	From 2007
Germany	Regional 2008, Universal 2013	Rotarix/RotaTeq	Medium	100	From 2007
Greece	Dec 2011	Rotarix/RotaTeq	Medium	100 (75% 2011–2021)	From 2009
Hungary	None	NA	NA	NA	From 2007
Italy	Regional 2013	Rotarix/RotaTeq	Medium	100	From 2007
Lithuania	2018	RotaTeq	Medium	100	2008–2013
Netherlands	None	NA	NA	NA	2007–2018
Slovenia	None (private)	Rotarix/RotaTeq	Low	0	From 2007
Spain	Risk group 2019 (pre-term infants) and private	Rotarix/RotaTeq	Medium	0	From 2007
Sweden	Regional 2014, Universal 2019	Rotarix	High	100	From 2007
United Kingdom	2013	Rotarix	High	100	From 2007

NA: not applicable.

^a^ Lithuania, Bulgaria and the Netherlands are no longer members of EuroRotaNet.

^b^ High > 80% for full schedule, medium 40–79%, low < 40% (data adapted from [9,35,36).

### Appraisal of rotavirus surveillance systems with focus on the European Rotavirus Network

The EuroRotaNet surveillance network has generated one of the largest datasets of rotavirus genotypes globally. The use of standardised typing methods and external quality assessment (EQA) systems allows for comparison and analysis of rotavirus strains across EuroRotaNet European countries and laboratories. An EQA is conducted yearly, organised by one of the EuroRotaNet partner laboratories and consists of a distribution of blinded panels of well-characterised wild-type rotavirus strains, including vaccine-type and mixed strains.

### Changes in molecular epidemiology identified through the European Rotavirus Network

**Figure 2:**
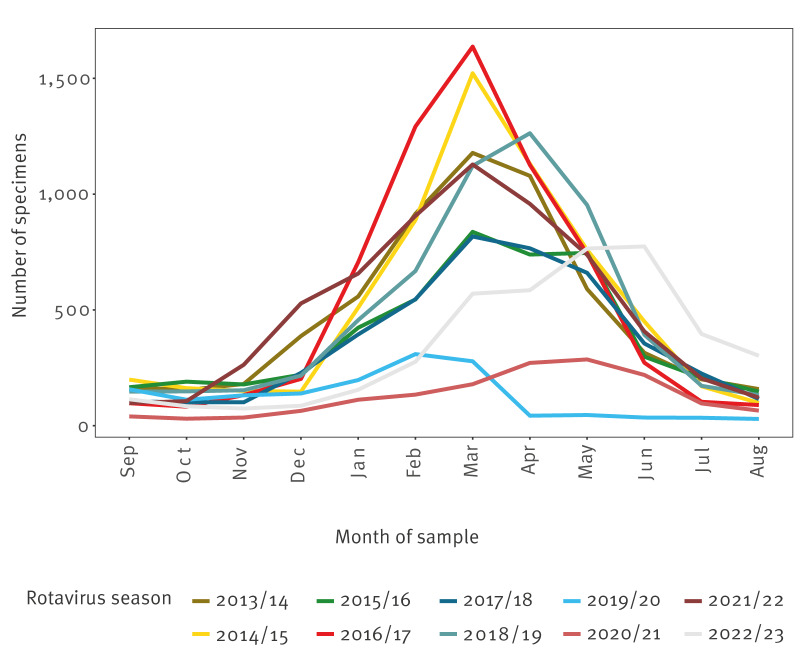
Temporal distribution of typed rotavirus specimens, all current European Rotavirus Network countries, September 2013–August 2023

As samples in EuroRotaNet are not subject to age restrictions, we are able to observe a distinct difference in the age distribution of particular rotavirus strains in the absence of vaccination. For instance, G2P[4] is more likely to be seen in older age groups [17,19]. The peak of the western European rotavirus season is normally in late winter/spring (Figure 2) and is often dominated by disease in children younger than 2 years caused by one dominant genotype, whereas there is greater genotype diversity out of season, mainly in older children and adults [17,19]. These findings suggest that natural immunity to some strains may be less complete or shorter lived, and this can have important implications for understanding and quantifying vaccine impact in the long term.

Countries included: Austria, Denmark, Finland, France, Germany, Greece, Italy, Slovenia, Spain, Sweden and the United Kingdom.

After the introduction of universal rotavirus vaccination with Rotarix in the UK (July 2013), in the pre-vaccine period September 2007 to August 2013, G1P[8] was the overall predominant rotavirus genotype (55%) [17]. In the first surveillance year of the vaccine era in 2013/2014, G1P[8]-type viruses appeared to become less dominant overall, accounting for 35% of genotyped samples [17], followed by a relative increase in G2P[4] strains from 2014/15 according to EuroRotaNet data [20]. In addition, when Belgium, Germany and Sweden introduced Rotarix into their national childhood immunisation programmes, the relative prevalence of G1P[8] declined and there was a rise in the proportion of G2P[4] strains detected [21]. Similar changes have been seen in temperate regions outside Europe, such as in Malawi and Brazil, after introducing Rotarix [22,23]. The changes in relative G2[P4] genotype dominance and the absolute decline in G1P[8] strains in EuroRotaNet countries and in studies from Malawi and Brazil may indicate vaccine selection pressures on genotypes with lower heterotypic vaccine effectiveness [24].

Nevertheless, shifts in genotype dominance need to be interpreted with care and in the context of substantially reduced rotavirus disease incidence. As in Finland, a country which introduced rotavirus vaccination in 2009 and exclusively uses the multivalent RotaTeq vaccine, G1P[8] was predominant in the pre-vaccine period (September 2006 to August 2009) and remained so until 2015/16, when the G9P[8] and G12P[8] strains dominated. This coincided with more widespread rotavirus vaccine use across Europe and concordant relative declines in the G1P[8] strain were observed across many EuroRotaNet countries, including countries without routine rotavirus vaccination.

A global systematic review of rotavirus genotypes showed higher G2P[4] prevalence immediately after vaccine introduction in vaccine-introducing settings relative to non-introducing settings [25]. However, to date, the higher prevalence of the G2P[4] strain has not been sustained, so any relative increase in detections of G2P[4] may be due to natural strain fluctuations or a transient effect of vaccination. If the emergence of G2P[4] as the dominant strain had been sustained, this would be concerning for vaccine impact because of the slightly lower effectiveness of Rotarix against the fully heterotypic G2P[4] genotype [24].

Although the data collected through the EuroRotaNet surveillance network is not designed to measure vaccine impact, in the UK there was a decline in the proportion and number of samples from infants younger than 12 months of age after vaccine introduction in 2013. In the pre-vaccine period, the median age of samples was 13 months compared with 19 months in the vaccine period [19]. Similar changes have been seen since vaccine introduction in 2013 in Germany and the 2014 pilot vaccination in Sweden, where the proportion of samples from children younger than 24 months of age declined post-vaccine introduction in both Germany and Sweden [20]. In Greece, age of cases increased with increasing vaccine coverage [26].

By being able to detect and monitor these changes in strain circulation in pre and post vaccine eras we can help inform the development of future rotavirus vaccines and monitor for vaccine pressure induced strain diversity.

### Identification and surveillance of vaccine-derived strains

Since the introduction of the Rotarix and RotaTeq vaccines, shedding of vaccine-derived rotavirus strains has been detected in children following vaccination [27]. In rare cases, vaccine-derived strains have been reported to cause gastrointestinal symptoms in both immunocompetent and immunocompromised children [27]. After the first dose of the vaccine, the peak time of shedding occurs between day 4 and 7, with prolonged shedding seen in immunocompromised children [28]. Although vaccine-derived strains have been detected in stool samples it does not mean that the gastroenteritis symptoms are caused by the vaccine-derived strain, as gastroenteritis could be caused by a non-rotavirus aetiology [27]. Countries who are members of EuroRotaNet employ molecular methods to detect vaccine-derived strains but Austria, Finland, Germany, Italy, Spain, Sweden and the UK are the only countries in the EuroRotaNet surveillance network to report vaccine-derived strains to the network [20].

Furthermore, in participating EuroRotaNet surveillance network countries, where individual vaccine status is available, surveillance can monitor genetic drift and potential VP4 and/or VP7 reassortment. This provides valuable information on whether these strains are widely circulating in the population or if they potentially represent direct transmission from a recently vaccinated infant/vaccine-strain shedder [20].

### Detection and monitoring of emerging strains

The EuroRotaNet surveillance network and GRSN allow the detection and monitoring of emerging rotavirus strains. Rotavirus strains emerging or reemerging after a period of absence in Europe between 2006 and 2021 included G8P[8], G8P[4], G12P[8], G9P[4] and equine-like G3P[8] strains [18,29]. Data from EuroRotaNet show that equine-like G3P[8] and G12P[8] strains have become well established in European countries [20]. Continued surveillance of genotypes across Europe is needed as was highlighted in 2020/21 when two samples from Finland included uncommon strains G1P[11] and G29P[4] which had not been previously detected in EuroRotaNet network countries, but G1P[11] had been previously detected in Estonia and India [20].

### Impact of the COVID-19 pandemic on rotavirus samples

**Figure 3:**
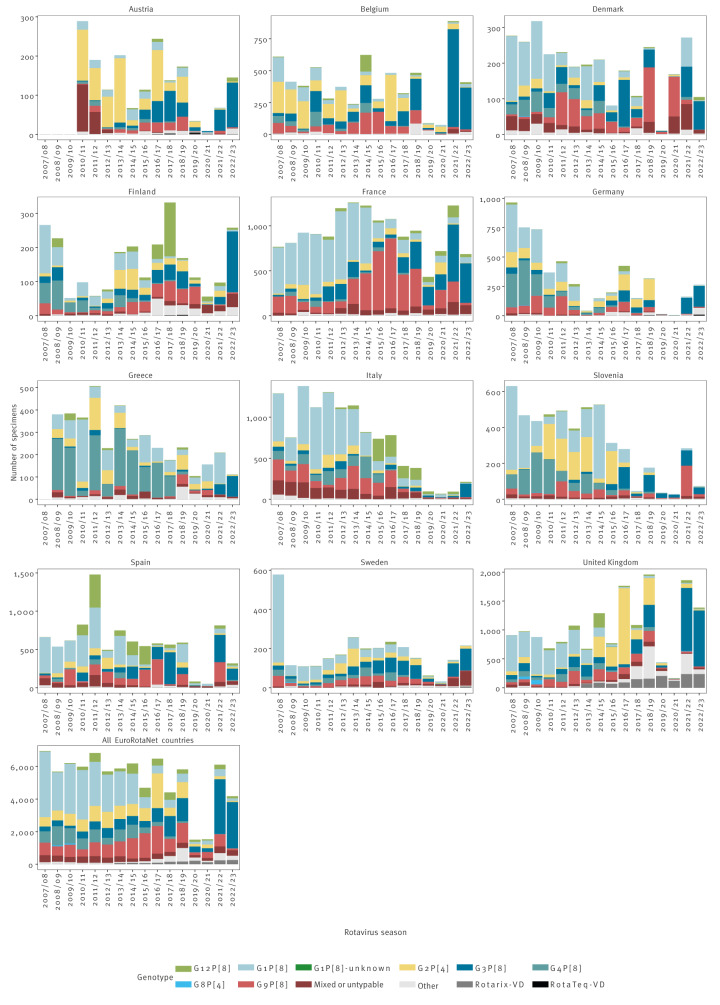
Temporal distribution of typed rotavirus specimens, all current European Rotavirus Network countries, September 2007–August 2023

In the 2019/20 (n = 1,510 samples in EuroRotaNet) and 2020/21 (n = 1,531 samples) rotavirus seasons, there was a substantial decrease in the number of samples typed and data submitted compared with in the previous 10 years (mean seasonal number of samples between 2009/10 and 2018/09 was 5,821) (Figure 3). This decrease in samples is likely to be a result of the COVID-19 pandemic, particularly since comparatively few samples were submitted during March and April 2020 when rotavirus detections would normally peak (Figure 2). The implementation of non-pharmaceutical interventions (NPIs) throughout the COVID-19 pandemic are thought to have reduced the transmission of rotavirus [30]. Furthermore, laboratory facilities and resources were re-allocated to COVID-19 testing and there were changes in health/healthcare seeking behaviours. However, through EuroRotaNet data submissions across all member countries (Figure 3) and surveillance reports from European countries, there is evidence that rotavirus has returned to pre-COVID-19 pandemic levels [31].

VD: vaccine derived.

### Strengths of rotavirus surveillance systems

Rotavirus surveillance systems provide infrastructure, collaborative expertise and potential financial support for countries involved. The WHO provides technical and managerial assistance, as well as financial support to countries eligible for Gavi, the Vaccine Alliance funding for rotavirus surveillance activities [32].

Surveillance systems provide countries with common guidelines on how to perform molecular assays, allowing for consistency between countries and quality assessment.

The EuroRotaNet surveillance network provides a sustainable model for rotavirus surveillance. It requires low investment and little infrastructure from member countries as network activities are funded by the collaborating institutes and industry, and only samples positive for rotavirus are typed using PCR. While there is a prescribed minimum dataset for each member institute to upload, it is achievable. This means the EuroRotaNet surveillance system can be used in both countries with different levels of public health and laboratory infrastructure.

The EuroRotaNet surveillance network is not prescriptive about which country-specific organisations need be involved, acknowledging the variance in public, private and academic funding and infrastructure used in rotavirus surveillance in each country. This means that the network has representatives from academia, healthcare, government-led public health/health protection and industry. Sharing of data and expert collaborations can further strengthen the scientific community and our understanding of rotavirus. Laboratories and scientists participating in these networks have generated data and expertise, which has supported the European Medicines Agency post-marketing requirements of rotavirus vaccines in Europe using relatively modest resources cost-effectively. Data generated through the network have supported and informed the recommendation for national vaccine introduction in Germany in 2013 and in France in 2022 [11], and contributed expert opinion on rotavirus vaccines for the European Centre for Disease Prevention and Control and the Netherlands Ministry of Health [33,34].

### Limitations of the European Rotavirus Network surveillance system

The EuroRotaNet surveillance network has limitations. Most countries within EuroRotaNet cannot link vaccination records with the laboratory data. Primarily, this is because most countries lack a shared clinical care record accessible to laboratories and because confirming vaccine status is not typically required as part of acute clinical care.

Diagnostic testing procedures also differ between countries and may change over time as more countries employ molecular methods. It is also not possible to report disease incidence as samples submitted to the surveillance systems represent only those samples referred for typing. The cases submitted to networks such as EuroRotaNet are likely to only represent moderate to severe cases as rotavirus is not a notifiable disease in most countries, and for many children symptoms are self-limiting and will resolve without seeking healthcare professionals.

## Conclusion

Continuous surveillance of rotavirus strains is important to improve our understanding of rotavirus evolution and genotype distribution, especially since there is evidence of slightly lower heterotypic vaccine effectiveness for Rotarix in middle- and high-income countries. The EuroRotaNet surveillance network provides a model for a pragmatic, sustainable, low input but high output surveillance. It allows for partnerships between public health departments, industry and academic institutions, strengthening the scientific community and our understanding of rotavirus strains. Rotavirus strain surveillance data are being used to inform public health strategies such as vaccination programmes.

## Data Availability

Aggregated data are available through EuroRotaNet reports (www.eurorotanet.com) and on a case-by-case request to the network.
